# Selection Mechanisms Underlying High Impact Biomedical Research - A Qualitative Analysis and Causal Model

**DOI:** 10.1371/journal.pone.0010535

**Published:** 2010-05-07

**Authors:** Hilary Zelko, Guilherme Roberto Zammar, Ana Paula Bonilauri Ferreira, Amruta Phadtare, Jatin Shah, Ricardo Pietrobon

**Affiliations:** 1 Department of Surgery, Duke University, Durham, North Carolina, United States of America; 2 Research on Research Group, Duke University, Durham, North Carolina, United States of America; 3 Pontifícia Universidade Católica do Paraná (PUCPR), Curitiba, Paraná, Brazil; 4 University of Joinville (UNIVILLE), Joinville, Brazil; 5 Kalpavriksha Healthcare and Research, Thane, Maharashtra, India; 6 Duke NUS Graduate Medical School, Singapore, Singapore; 7 Duke University, Durham, North Carolina, United States of America; University of East Piedmont, Italy

## Abstract

**Background:**

Although scientific innovation has been a long-standing topic of interest for historians, philosophers and cognitive scientists, few studies in biomedical research have examined from researchers' perspectives how high impact publications are developed and why they are consistently produced by a small group of researchers. Our objective was therefore to interview a group of researchers with a track record of high impact publications to explore what mechanism they believe contribute to the generation of high impact publications.

**Methodology/Principal Findings:**

Researchers were located in universities all over the globe and interviews were conducted by phone. All interviews were transcribed using standard qualitative methods. A Grounded Theory approach was used to code each transcript, later aggregating concept and categories into overarching explanation model. The model was then translated into a System Dynamics mathematical model to represent its structure and behavior. Five emerging themes were found in our study. First, researchers used heuristics or rules of thumb that came naturally to them. Second, these heuristics were reinforced by positive feedback from their peers and mentors. Third, good communication skills allowed researchers to provide feedback to their peers, thus closing a positive feedback loop. Fourth, researchers exhibited a number of psychological attributes such as curiosity or open-mindedness that constantly motivated them, even when faced with discouraging situations. Fifth, the system is dominated by randomness and serendipity and is far from a linear and predictable environment. Some researchers, however, took advantage of this randomness by incorporating mechanisms that would allow them to benefit from random findings. The aggregation of these themes into a policy model represented the overall expected behavior of publications and their impact achieved by high impact researchers.

**Conclusions:**

The proposed selection mechanism provides insights that can be translated into research coaching programs as well as research policy models to optimize the introduction of high impact research at a broad scale among institutional and governmental agencies.

## Introduction

The history of science is filled with stories of innovations and “high impact” ideas that transform established ways of thinking and standard practices. Historically, the generation of these ideas has interested primarily scientists themselves, philosophers and historians of science. Today, however, the audience is broader. Scientific innovation is not only important to individual researchers and academic institutions whose careers, missions and reputations rely on meaningful and high quality research, but also to funding agencies and foundations that are constantly deciding about research priorities and resource allocation. Increased competition for research funding and greater pressure to generate meaningful results and practical applications is driving a greater emphasis on innovation among both producers and consumers of scientific research. Despite this growing interest and an explosion of quantitative efforts (i.e. citation indices and analyses) aimed at evaluating research performance [1], [2], few studies have examined from the researchers perspective how impact is achieved.

In Bruno Latour's comprehensive examination of laboratory life he reveals that scientific findings/facts are neither simple flashes of brilliant intuition nor purely logical deductions, but rather “social constructions” that emerge from the rather chaotic daily activities and interactions in the lab. He states, “we argue that both scientists and observers are routinely confronted by a seething mass of alternative interpretations. Despite participants well-ordered reconstructions and rationalizations, actual scientific practice entails the confrontation and negotiation of utter confusion” [3]. He proceeds to explain how these social processes of “confrontation and negotiation” are critical to the development of scientific ideas.

Using a different approach, Nancy Nersessian also explores processes by which scientific ideas are generated in her book “Creating Scientific Concepts”. Unlike Latour, Nersessian analyzes historical examples of conceptual change to focus on the underlying cognitive processes employed by scientists in generating new concepts. Though her focus is slightly different, Nersessian reaches a similar conclusion to Latour in her analysis of how scientific concepts arise. She states, “…conceptual innovation, like perfect orchids and flavorful grapes, emerges from lengthy, organic processes, and requires a combination of inherited and environmental conditions to bud and bloom and reach full development” [4].

While these studies provide important insights into the development of scientific ideas, they are broad in scope and don't address the question of why some scientists are able to consistently generate high impact ideas and publish at levels far beyond the average.

Studies of faculty research productivity among biomedical researchers have identified a number of individual and institutional characteristics that are associated with high levels of research productivity. For example, Bland et al [5] found that a combination of factors including individual motivation and passion for research as well as institutional characteristics such as having sufficient work time to conduct research, and having mentors or a network of colleagues involved with research were associated with higher publication rates. While these studies have made great strides in identifying variables related to productivity as measured by number of publications, they don't address questions about the techniques or methods researchers use to generate and publish high impact ideas in the first place.

The objective of this qualitative study is to investigate a group of biomedical researchers with an unusually high impact in their scientific fields, searching for consistent patterns of behavior over time or heuristics that could explain their success.

## Methods

Our study was approved by the Institutional Review Board of the Duke University Medical Center. All potential participants were initially approached by email and then sent an electronic copy of the consent form, which they returned by fax or emailed a scanned copy. At the beginning of each interview, an additional explanation about the study was provided, with an emphasis on their privacy and additional questions if any, were answered. Finally, in order to ensure confidentiality, all subjects were given the opportunity to review our results, with any issues related to confidentiality being immediately addressed.

### Subjects

Because our study involves a very selected study sample, and since our inclusion criteria could lead to identification of subjects, only partial information about inclusion criteria are provided. For the purpose of this study, high impact researchers were defined as those having five or more peer reviewed publications with an impact of over 300 or an h-index [6] of 50 or more. Additionally since we are conducting a parallel study evaluating researchers who were considered as of high throughput (high number of publications), researchers meeting both criteria and already enrolled in the high throughout study were excluded. Individual universities around the world were selected using a convenience sample, school names not being released to protect the privacy of our subjects.

### Interview procedure and transcription

Since some of the subjects were located in remote cities, we opted for conducting all interviews by telephone to standardize the procedure across all of them. One of us (RP) conducted the interviews by telephone, each of which lasted between 20 and 40 minutes. All interviews were digitally recorded using call graph [7] and then transcribed by trained personnel following standard qualitative methods [8]–[10].

Following the method described under grounded theory for qualitative studies, we used a semistructured script to guide the conduct of qualitative interviews. During each interview we tried to understand the views and ideas held by each subject in relation to the high impact of their scientific work. After each interview, we held a discussion amongst the members of the research team (HZ, JS, RP) for an initial evaluation of the interviews content as well as to make modifications to the interview script as necessary. This constant comparison between the responses in an ongoing interview with the responses from previous subjects allowed us to remain close to the concepts encountered while refraining us from adding our personal assumptions. Moreover broad themes were identified during each interview and explored in the subsequent interviews in an effort to validate/invalidate the themes encountered. A final script of the interview is provided in Appendix S1.

### Analysis

Interviews were transcribed and read independently by one of us (HZ), while the two other authors (RP, JS) discussed the emerging themes with her. All interviews were analyzed by one researcher (HZ) using a combination of manual and software-guided coding [11], and discussed with two other researchers (JS and RP). Ambiguities were resolved through discussion. Categories were reduced to major themes through ongoing discussion between researchers and the re-reading of transcripts [12]. All involved researchers were familiar with qualitative research methods and Grounded Theory. Grounded theory attempts to generate themes from the interview transcripts, which are called emerging themes. This method is in contrast with the usual scientific method where a hypothesis is first generated and then tests are performed in an attempt to falsify it. Grounded theory was conducted in our study by coding the transcripted interviews using a set of codes. These codes were then grouped into overarching concepts, categories, and finally a model that attempts to explain the underlying coherence [13], [14]. Of essential importance to this study, all participating researchers have a direct interest in the study of research processes through the Research on Research Group at Duke University [15] and are familiar with the literature on research innovation. Given this familiarity, although we started planning to use a Grounded Theory approach, it was evident in our first three interviews that we were attempting to find a “universal method of innovation.” This initial attempt, which turned out to fail as will be detailed in the results section, had a significant influence on our results since we had to completely change our approach and really make our themes emerge from the content of the interviews rather than trying to force a non-existing theme that was coming from assumptions in the literature.

### Triangulation

In order to validate our results, we used a triangulation mechanism by comparing it to the observation of subject's publications prior to the interview in the context of the content of their interviews. During these observations we attempted to make sense of their comments in relation to the temporal evolution of their publications. This facilitated the contextualization of the interview. For example, when a subject told us that a certain publication was important since it was elaborated following a certain heuristic, we could then check the content of that publication, which other references immediately followed it, and for how long it had been cited. All citation analyses were conducted using a mix of the ISI Web of Knowledge database [16] and Google Scholar. [17]

#### Respondent validation

Respondent validation was achieved by submitting a two-page summary with our main findings to all study subjects while asking for their input. Their input was then coded and either incorporated into our emerging themes or our list of negative cases. Negative cases were listed whenever some evidence was found that our emerging themes presented exceptions among our interviews, the nature of this disagreement being explained and exemplified through selected quotes.

### Modeling strategy

In order to better understand the relationship among the emerging themes, we developed a Systems Dynamic model connecting all emerging themes. System Dynamics (SD) is an approach to help understand the behavior of complex systems over time [18], complex systems broadly defined as a set of independent elements that interact with each other in a stable way. When we face complex situations with an uncertain number of parameters that are difficult to quantify, our mental models may lose their structured view of the problem, its key aspects and possible solutions. In this way, SD models help us to understand the entire process and identify points to intervene [19]. Originally developed to help corporate managers improve their understanding of industrial processes, SD is now being used throughout public and private sectors for policy analysis and design [20].

After obtaining information from the qualitative analysis, we collected expert opinion from a small panel (n = 4) of researchers and research policy experts in order to identify the behavior of the system in question from a broader perspective. Based on the literature review and expert opinion, we first created a preliminary version of the causal model using the software Vensim DSS 5.9c [21]. This preliminary model was created based on two archetypal structures: “limits to success” and “success to the successful”. [22]. These archetypal structures represents behavior patterns, such as an element limiting the growth of a system (limits to success) or rich getting richer and poor getting poorer (success to the successful). It was populated with data obtained from the qualitative analysis of our study, generating the final version of the model. Model validity was considered if the overall time trend behavior represented by the collection of emerging themes and their relationships were to generate a behavior plausible with the average scientific and impact productivity obtained by most subjects.

## Results

Among our interviewees, high impact emerged from two primary sources. First, impact could be generated from new ideas that either created a new field or significantly changed the direction of an existing field. Second, impact could also arise from ideas that lead to practical applications that changed clinical or public health care practices. For example, several researchers stated that the high impact of their publications was related to the introduction of new ideas that were “first in the field” or opened up a “bottleneck” in a field. One researcher noted, “…for me impact is not just having a discovery but in fact moving it to next place of research…” Other researchers focused on practical applications of research in order to change clinical or public health practice. Regardless of whether they were oriented toward expanding the scientific knowledge base or applying that knowledge to healthcare practice, five main themes emerged from our study: (1) researchers apply heuristics or rules of thumb which guide the development of new ideas; (2) feedback from mentors and peers reinforces the continued application of these heuristics; (3) feedback to peers (in the form of presentations/publications) ensures the recognition and diffusion of ideas; (4) psychological attributes such as curiosity, open-mindedness, and flexibility enhance researchers receptiveness to feedback.

### Emerging Themes

#### Heuristics

It was apparent from our study that researchers use a variety of “rules of thumb” or heuristics to guide them in pursuing new ideas, and usually they could identify one main heuristic that made them thrive among their peers. Some researchers could identify this heuristic immediately, while for others this heuristic was not evident until some time into the interview. These heuristics were typically not something that researchers themselves planned to use so that high impact could be generated, but instead it was simply something that they were “good at” and that happened to generate impact when used in a research project.

Although each and every researcher had a main heuristic, specific heuristics varied broadly across researchers and were hardly comparable. For example some researchers emphasized the heuristic of throwing a broad net of experiments and then finding innovation through a trial and error process, only some of which resulted in unexpected or novel results. One researcher commented, “*Its a lot of elbow grease, none of these were brilliantly planned experiments, where you did only one or two experiments, each one of them yielding brilliant results. No ours was doing hundreds of experiments and then occasionally picking out the great ones.*” And another noted, “…a*fter trying out different things and then suddenly something works and you run with what works*.” The iterative nature of the trial and error process was expressed in the following quote, “…*basically we were going continuously back and forth between the laboratory and clinic, always attacking newly recognized problems from the clinic then in the laboratory and as you well know, in research, more often than not, you are headed down a dead-end track but by doing many of these journeys we always managed to find the final throughway, so to speak,* toward *the next application*.”

In complete contrast, other researchers focused on heuristics relying on a more planned or step-by-step approach. For example one researcher noted that in his group they plan carefully ahead of time with statisticians in an effort to keep experiments simple and straightforward. “*…we sit down with them [statisticians], we plan readout with them, you know primary objective and then secondary ones and stopping rules and …we try to keep the readout very very simple. Black and white, if you wish, or something close to it. If you have a very complex experiment we, I believe at least, it is bound to really complex answers. It doesn't really help you all that much most of the time.*” Other researchers explain that they approach problems “step-by-step” - “we tackle the perceived problems one by one”. Another respondent explained, “Once we accomplished that 1st step, it was startling the new found problems one by one basis.” Other heuristics were related to the identification of research questions. For example, identifying interesting or important research questions was associated with knowing the research literature really well, identifying knowledge gaps in the field or aiming to resolve contradictions or address limitations in previous studies. One researcher remarked, “*…sometimes I have just read the literature and found it to be contradictory, or not satisfactory and decided to have a large cohort study, so that I could look at it again and perhaps throw some light on that, and that has turned out to be useful.*” While another said, “*Key thing is to know the literature and to know the field that you are in*.”

A key feature of important research questions is the potential for changing a way of thinking or practice. “*I want to focus on… what I would consider to be important, interesting question and so what I always ask myself and my students is what would you or someone do with that data, how it is going to be, what's the next step, how would it change somebody's way of thinking or somebody's way of practice and if you can't answer that, then it is better to do a different topic.*”

Another heuristic expressed by our study subjects was the use of “out of the box” thinking and challenging conventional wisdom, allowing them to approach research questions from different perspectives and focus on practical applications of research. One researcher stated. “*I enjoy …the iconoclastic or out of the box ideas and… that's always been something that intellectually has been satisfying to me*.” He explained that one of his initial breakthroughs came about because he was able to utilize a completely different perspective than the current thinking. Another researcher noted that she often “disagreed with conventional wisdom” which lead to high impact ideas and a different explained that what's most important to have genuine impact is to “change people's views”. Other researchers explained that their clinical background/perspective significantly informed their research work and pushed them to pursue ideas that would have practical applications - changing clinical or public health practice. “*I am a clinician at heart so my outlook is colored by this…is it [research question] related to human disease and is there a potential therapy emerging from it…*” Another researcher emphasized that his focus on practical applications of research were fundamental in achieving impact, “*I have been focused on the application of findings in firm policies and prevention strategies. I guess…that translates to studies and contributions that can really be applied in the real world and inform clinical and public health practices and policies. And from my perspective that is the way impact counts.*”

Focusing on practical applications was also considered to be important in shaping the kinds of topics that were pursued. “*certainly some of my work is purely building a knowledge base but had a major commitment to the applied use of the knowledge, which probably then influences in some part the sorts of topics I take on*..”

In sum, each and every one of our interviewed researchers could point to one main heuristic that they followed, and that usually made them unique among their peers. This uniqueness came sometimes from others not being able to reproduce the heuristics, but more often from the researchers being innately good at it. For example, one of our researchers noticed that his data collection for large registries was so detailed and took so long that other researchers would hardly be able to accomplish what he accomplished. In other words, his heuristics made him unique, and this uniqueness guaranteed his high impact among his peers. These heuristics were not created by design, but were simply actions that they were proficient with and enjoyed using. But simply having these heuristics cannot explain how they were selected over time. For this selection to happen, researchers had to have a feedback mechanism, which leads us to our next emerging theme.

#### Feedback from mentors and peers contributes to the selection and development of heuristics

Another theme in our study was the important role of feedback which comes from mentors and peers in both the maintenance of a certain heuristic as well as its continued development. This heuristic selection process started early on in their careers when feedback from mentors served as a source of motivation and instruction (“I picked up from my mentors that guidance in terms of ways of thinking and guidance and emphasis and vigor.”), but they also shaped the development of researchers' heuristics. For example, one researcher noted that her mentors were essential for conferring critical thinking skills which allowed her not only to understand the limitations of other studies, but also be self-critical of her own results. These skills were critical for her heuristic which consisted of knowing the research literature and identifying gaps or contradictions in the literature that would become the source of new project ideas. Another stated that his mentor “pointed out…the critical nature of trying to identify and address the important problem”, which became a key component of his heuristic of pursuing important research questions. In another case, a mentor's lessons in single-mindedness carried over into a heuristic emphasizing a highly focused, systematic/step-by-step approach to research. Of importance, what she identified as her main heuristic, namely a highly degree of attention to one of the central methodological areas in her field, was constantly re-inforced as a “good” skill to have. Another researcher noted that sometimes a mentor provided an example of what not to do. Mentors were not always a primary source of feedback or a central contributor to success. Although some of our subjects credited mentors with providing essential guidance and skills early on that contributed to their success, others placed greater emphasis on peers. Feedback from peers served to reinforce the significance or the limitations of ideas (i.e. critiques) and ensure the clarity of the message. “*I try very hard to be self critical but it [feedback from colleagues] is helpful to see limitations. But I think the biggest help is in terms of clarity of the message, if I present it to peers and they don't get it then it means that I am not explaining it well enough.*” Another person stated that feedback from junior colleagues ensured the application of new and highly sophisticated methodologies.

In sum, in addition to a set of heuristics, researchers in our study had these same heuristics both motivated and enhanced by feedback from others. This selection process made them successful within their scientific environments. However, at this point it was still unclear how exactly the scientific information they generated was being diffused to the scientific community at large, which brings us to the next emerging theme.

#### Feedback to peers contributes to diffusion of ideas

Feedback *to* peers consisted primarily in the presentation of new ideas through publications and scientific presentations. Most researchers stated that they spent a significant amount of time and energy working on the presentation of their ideas because of its importance in ensuring that ideas were clearly expressed and ultimately, recognized. One researcher remarked, “*I don't mind putting a lot of energy in to fine tuning, writing, and making sure that it [the message] is clear and logical and appealing for editors and readers….I have seen this a lot where poorly written papers don't get into good journals, even though they could have if they had been better written and better presented… there is a lot of good science out there that is essentially ignored because it is not presented well. History of science is full of examples of important discoveries that were just passed over. Usually people take home the message that they were passed over because scientists are just stuck in a rut or don't want to look at new paradigms, but a lot of times those important things were passed over to because they weren't well written, and they weren't clear, and they weren't persuasive.*” Another researcher explained that she spends a lot of time on the presentation of results in order to ensure they are accurate. “*I spend huge amount of time [on presentation], because…there are so many ways that you can be misled in study as they are cross-sectioners, confounders, effect modifiers that you haven't considered, you know there are just many many ways, where one looks like an obvious conclusion would turn out to be wrong.*” Other participants noted that part of having high impact is reaching a broad audience and writing results in a way that non-specialists can understand. “*We pay lot of attention to write up. We spent lot of time on doing the writing. Trying to introduce, why you did, what you did… The basic idea is to make it readable to an audience, which is not necessarily* all *scientists working in this field and also, if its clinical paper we want the [clinician] to be able to understand what we are trying to show… So we are trying to write in a way that gives a broader impact…and understood by a broader audience.*” Another study respondent emphasized that clarity in the presentation of ideas was critical for achieving high impact, “…*it's a fundamental component [of high impact] clear thinking, clear framing of the analysis, clear summarizing of the data in tables and figures and then clear writing that's without a doubt, fundamental*”.

In sum, although having a unique heuristic that is constantly enhanced by feedback from others, the end product of a high impact researcher still has to be communicated in the most clear and convincing possible manner. In a way, this communication can be perceived as a way to pass the information generated by the heuristic to others, a communication channel that will generate the necessary feedback to keep nurturing the constant development of the heuristic. What is now missing in this chain of emerging themes is the actual engine to keep it in motion. In other words, what motivates researchers to keep doing what they do despite the multiple adversities and problems that they find in their work. This gap is filled by our next emerging theme.

#### Psychological Attributes

A number of psychological attributes were identified by the researchers as pre-requisites for high impact work. For example, many researchers stated that curiosity was an important characteristic that lead them to explore important research questions. One researcher noted, … *it was a combination of a voracious reader and a very curious person*”, while another said, “*I didn't feel any external pressure to write papers because I wanted to learn these things and if I found something I wanted to get it out there.*” Also, curiosity propelled some researchers undergo training in other fields (i.e. mathematics and medicine) or pursue research areas outside of that in which they had been trained. One researcher stated, “*I had this feeling of curiosity, I wanted to do something more than whats in the text book… So my first step was I went into basic biology for about three years.*” Then once in that field, his curiosity lead him to question whether the experiments would have any real impact on human disease which then spurred him to investigate new areas. On the other hand, one researcher noted that he pursued ideas not simply for the sake of curiosity, but more importantly because they could have some practical application to clinical or public health practice. “*I know some scientists just filled with curiosity, why is it when you cook eggs they turn brown. This is not a question that would ever engage my energy…I am more motivated by how this would help people*…”

Other attributes that were emphasized were open-mindedness, dissatisfaction with the status quo and persistence. One researcher captured all of these attributes in his statement, “…*if I were to label things that maybe potentially learnable by somebody it would be the capacity to listen and to be curious and to be open minded and be ready to challenge and have an inclination to challenge and to be critical.*”

Most of our subjects mentioned that they had developed these attributes while there were in school or during the early phases of their in the careers. Keeping one's eyes open and an open-mind is important for recognizing the significance of unusual or unexpected results as well as pursuing opportunities that might arise serendipitously. “…*you try a lot and keep an open mind and you see the gold nuggets in the sand… do a lot of work*.” Persistence was critical for the pursuit of unusual and seemingly “dead-end” ideas as well as following through with the “hard work” of research. One respondent stated, “*You are asking me why have I never been later than 6 am in my office for 25 years and the reason for that is that my driver is a personal dissatisfaction with the status quo*.” Another said, “…*once I tackled the problem, I want to finish it and I want to finish it … well … having all pieces in place.*” Other researchers noted that the pursue ideas even when they (or others) are doubtful about the potential results. One researcher remarked, “*When we did design the actual study… we had very dim hopes actually because the results in the laboratory… really didn't make all that much sense. They were always false negative and false positive. So it was actually very discouraging…*” Another person stated, “*…we had a novel technology in the laboratory that nobody thought would be really helpful in patients. We went ahead anyways…*”

In sum, irrespective of the heuristic they use, the type of positive feedback they receive, or the way they communicate the results of their heuristics, researchers achieving a high impact are highly self-motivated, curious, and enthusiastic about their work. This sense of curiosity about discovering is what keeps them constantly using the heuristic over and over, which in turn provides them with more and more positive feedback, ultimately turning itself into a positive feedback system. But this system is certainly not linear, and this randomness can sometimes work in their favor or against them. This serendipity is the final theme found in our study.

#### Randomness and serendipity

While many of the researchers in our study indicated that they could immediately recognize when their findings would have a great impact, they often attributed their achievement of high impact to luck, serendipity or being “in the right place at the right time” rather than any particular cognitive/behavioral or methodological factors that would consistently lead them to new ideas/results. For example, when asked how they were able to so consistently achieve such high impact in their work, several researchers stated that they seized opportunities to work on projects that came to them “serendipitously” or because they happened to be in “the right place at the right time” when an opportunity emerged - “*It was being in the right place at the right time and seizing right opportunity*…” and another said “*Mostly it was luck. I was in a right place at right time*”. Another scientist described how opportunities came his way, “..*so those things came across my desk I got interested in them but I wasn't sitting around spending time thinking every month what should I do to have a bigger impact*.” Another said, “*For me it was nothing rational, not planned*.”

Others noted that their research produced unexpected findings as result of trying different experiments even when they were doubtful about potential results. For example, one researcher, when describing one of his early breakthroughs stated, “*When we did design the actual study… we had very dim hopes actually because the results in the laboratory… really didn't make all that much sense. They were always false negative and false positive. So it was actually very discouraging and recalling that night that we decided on an in-vivo experiment, we had the choice between [giving up] … or doing one last study and we decided on one last study…and then when the study was done, it was stunning… and we knew there was very important information, because it was the very first time anybody had shown that an in-vitro test, even a bad one, predicted outcome of a transplant*.” Speaking more generally about how he achieved high impact, another researcher stated “…*after trying out different things and then suddenly something works and you run with what works” and that he “…had the good luck to define something that clicked.*”

### Policy Model

**Figure 1:**
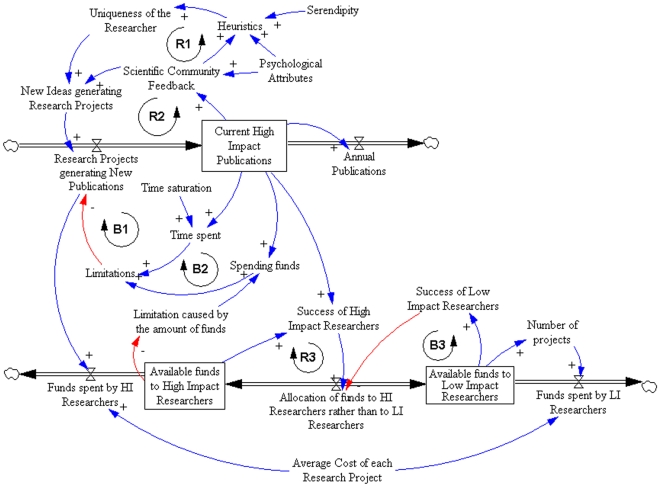
System Dynamics model demonstrating the relationship among all emerging themes found in our study.

Instead of selecting one approach (heuristic) that leads to high impact publications and differentiates them as researchers from other members in the community, high impact researchers seem to use one innate ability they naturally have. This innate ability provides them with a natural fit to the social scientific environment, and differentiates them from others, makes them more fit to succeed in this environment from the very beginning of their careers. These heuristics vary widely and do not seem to have a common pattern across researchers, at least within our study sample. Heuristics were certainly helped by serendipity. Because this innate heuristic differentiates them and makes them fit and more likely to succeed, they will re-use this heuristic throughout their careers, either within a single field, or carrying the heuristic to another field. The heuristic also reinforces itself, since a successful use of the heuristic (leading to a high impact publication) will gain support from peers, thus making the researcher use it over and over again. Another positive feedback loop is the one provided by their personality traits identified in our model (Figure 1). Although we have not followed a control group with consistent low impact rates, it is likely that this positive feedback loop would not be present for them. When carrying this heuristic to another field, they will specifically select areas where the heuristic could once again provide them with a fit advantage, thus reproducing the same pattern of success obtained in the previous field. For example, a researcher's ability to carefully create detailed databases will make this unique heuristic a differentiating factor. This pattern of an innate heuristic that provides better fit and greater likelihood of success or “survival” within a scientific community presents an interesting analogy with selection or evolutionary processes.

In our model (Figure 1), these patterns are represented by the following feedback loops named as R1, R2, R3 (Reinforcing loops) and B1, B2 and B3 (Balancing loops). The first reinforcing loop (R1) describes the interactions between the researcher with their innate heuristics while being influenced by psychological attributes as well as the feedback from their peers. These heuristics make researchers unique among their peers, ultimately making them more capable to generate new ideas that culminate in innovative research projects. More research projects generate more high impact publications, then generating the positive feedback by peers and mentors, and ultimately reinforcing the heuristics themselves as reinforcing loop.

**Figure 2:**
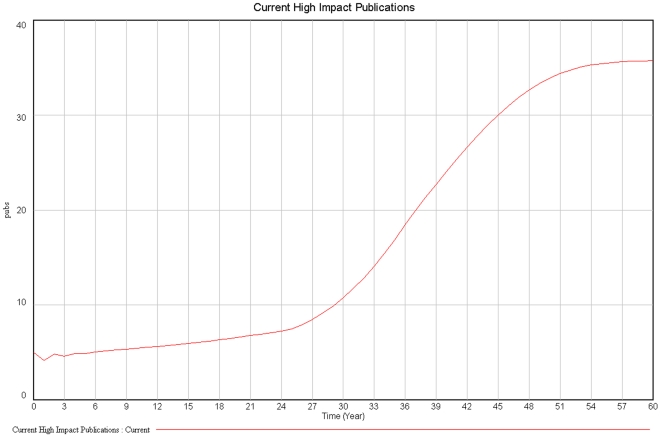
High Impact Publications over time.

The second reinforcing loop (R2) reflects the influence of publications on peers, leading to the generation of new ideas, research projects and publications. If only the R2 loop were to be considered, there would be no limits to an exponential growth in scientific production. Instead, this growth is limited by time (loop B1) and funding constraints (loop B2). The more a researcher generates, more time and funding are spent. The more time and funding are spent, fewer research projects will be viable, ultimately placing a plateau to the overall number of publications that are possible within this system (Figure 2). This interaction between loops thus characterizes loop R3 as a limit to the growth of the entire system.

**Figure 3:**
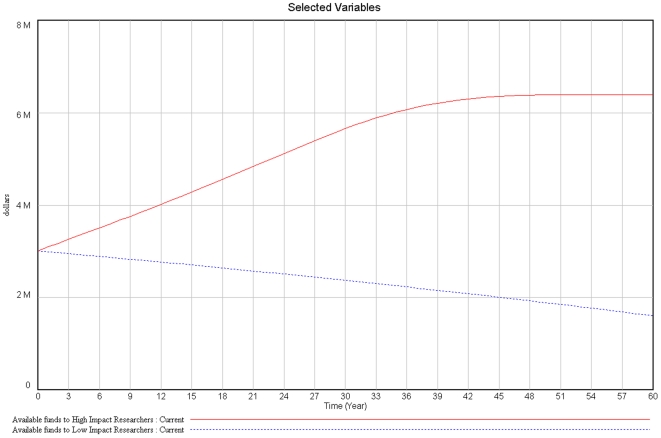
Available funds to ‘High Impact Researchers’ versus ‘Low Impact Researchers’ over time.

In addition, the more successful the researcher is, the more funding becomes available, decreasing the limitation caused by funding and increasing the scientific production and the respective success of the individual researcher. In contrast, low impact researchers will have more difficulty obtaining funds, therefore decreasing their scientific productivity and consequently their success, making funding even more difficult (loop B3). In our model, this scenario is simulated with two researchers, one with high impact and the other with low impact. Both start their career with the same amount of resources. The high impact researcher will generate high impact publications, making resources grow. The low impact researcher will generate low impact publications, consuming resources until they are extinguished (Figure 3).

## Discussion

To our knowledge, this is the first study conducting a qualitative evaluation of high impact biomedical researchers from different institutions around the globe. Through our qualitative study we uncovered 4 major themes that serve as the source of high impact in scientific research: Heuristics, feedback from peers, feedback to peers, psychological attributes, and randomness and serendipity. Our results demonstrate that high impact biomedical research is highly complex, emerging not simply for individual genius or pure luck, but rather, from a dynamic selection process involving cognitive and environmental components. In this sense our argument is similar to more anecdotal and popular works such as Malcolm Gladwell's ‘Outliers’ which argues that success is shaped as much be environment and opportunity as individual talent or hard work. However unlike Gladwell's work which points to traditionally sociological factors (i.e. class, age, education) we focus on cognitive and behavioral factors interacting with the environment. Specifically, we found that researchers employ heuristics that guide the development of research questions and the process of research. Feedback flowing both from and to the scientific community contribute to the development of continued application of successful heuristics. Psychological attributes such as open-mindedness, curiosity and persistence enhance researchers' receptiveness to feedback as well as, contribute to searches for new problem areas or opportunities. Finally, serendipity serves as a higher level selection mechanism that pushes forward new ideas and opportunities for further research. Once researchers produce their initial works of high impact, they typically enjoy greater access to resources and research opportunities, further reinforcing their cognitive and behavioral strategies, much like the Matthew effect (which proposed the phenomenon - rich get richer and poor get poorer) enhances the position of established scientists through reward and communications systems and leads to “the concentration of scientific resources and talent” [23]. As we will argue in this section, it is through the lenses of a selection paradigm that we can aggregate these themes and better understand why it is that high impact research seems to emanate from such a small group of researchers.

A heuristic consists of a set of rules [24] that guide individual scientists on what to do or not do [25]. Our findings indicate that contrary to our initial hypotheses high impact researchers follow a process that bears an analogy to selection processes, although this analogy is not perfect. Instead of having a pre-defined plan for achieving high impact, these researchers use heuristics that are natural to them to find a unique niche within their field. This characteristic, which we call an impact heuristic, is transported with them when they either move to new projects or even when they move to other fields. When moving, they search for other problems or fields where their heuristic would make them more likely to be successful, ultimately creating a positive feedback loop. In addition to distinguishing them from others and giving them a competitive advantage in research, these heuristics did not follow a pattern but were scattered across a wide range of personal and group characteristics. Our findings resonate with Feyerabends argument that successful research seldom follows a standard pattern and it utilizes different methods (‘tricks’) at different points of time. Researchers (‘movers’) are seldom aware of 1. the moves (methods) that advance scientific research and 2. the standards that determine what counts as advances [24]. Given the wide diversity of methods across researchers even when considering one single field [26], the idea of researchers thinking rationally about methods to falsify theories dissolved to make way for a much more human-centric approach [27], [3]. Our findings in terms of transport of impact heuristic finds support from Lakatos [28] who argued that heuristic rules were not obligatory and once could reformulate their pattern of thinking (heuristic) thus leading to possibilities of methodological invention [29]. Heuristics therefore play an important role in making researchers unique within their scientific environment, and confer them with an advantage over their peers that will ultimately enhance their chances of success in their careers.

In a selection model, the unique features of a living creature have to be reinforced by its environment. The same effect was found to occur in relation to the heuristics in our study. Analysis of subject responses revealed that feedback from peers, mentors and scientific community contributed extensively to the maintenance and further development of their heuristics and ultimately to their high impact. As has been pointed in other educational processes, feedback from peers contributed to the improvement of students' skills over time [30]–[32]. Similar results were obtained in business environments [33], thus characterizing feedback as a social mechanism that allows individuals to adapt to social norms. Given that science is a social environment characterized by traditions, rituals, power, and social norms [34], it is no surprise feedback loops are a constant in this system. The same applies to feedback systems where researchers are communicating to their peers, which allows for an enhancement of learning processes by means of reflection, analysis and diplomatic criticism [35]. These feedback mechanisms justify our choice to create a policy model using System Dynamics, which is concerned with the study of complex behavior in a system resulting from dynamic interactions (‘feedback’) among its components. [36], [37].

While it is often thought that innovative researchers have more “creative” personalities and thought processes than most people, recent research on creativity in psychology suggests that innovations emerge from more ordinary thought processes than previously imagined [38]. For example, Weisberg desribes how Watson and Crick “adopted and extended pre-existing ideas” in developing their DNA model. This does not mean that there are not certain characteristics or attributes that may be associated with innovation and creativity. For example, J.P Guilford identified “divergent thinking” as a core feature of creative thinking which allows for production of out-of-the ordinary ideas [38]. More recently, Simonton has suggested that certain dispositional traits are present in highly creative scientists such as an intellectual ability to generate many different associations or having a trait such as “…openness to experience, including defocused attention and receptiveness to novelty, variety, complexity and even ambiguity” [39]. Our results, which identify several psychological attributes associated high impact researchers such as open-mindedness, flexibility, curiosity and dissatisfaction with the *status quo* do suggest that certain psychological attributes may predispose researchers to develop novel ideas, pursue unusual research paths, or recognize the significance of unusual findings. However, psychological attributes alone cannot explain why some researchers are consistently able to achieve high impact, while most do not. Rather than serve as determinants of innovation, we suggest that these psychological attributes act as facilitators that enhance researchers' receptiveness to both feedback and serendipity.

The idea that serendipity or chance is an important component of scientific innovation has been documented in historical and psychological studies of science [39], [40]. While its significance in the discovery process has been debated [41], it continues to be advocated by a majority of those studying scientific innovation [42]. Given the consistency with which the researchers in our study referred to serendipity, luck, or being in the right place at the right time, our results indicate that there is indeed a role for serendipity in scientific innovation. We find that rather than being a “cause” of scientific innovation, serendipity acts as the engine behind the selection mechanism, provides researchers with the variability that is necessary to generate innovation, both cognitively, i.e. generating innovative ideas, as well as circumstantially, i.e. generating opportunities to pursue novel ideas.

The idea of selection processes has been previous raised by authors in the Science and Technology Sciences community [43], [44]. Previous approaches, however, have focused on selection as applied to theories rather than the behavior of individual researchers as taken in our project. Criticisms have also been made through the argument that the mechanism at play in the selection of theories cannot be compared to natural selection since the latter is associated with random events, while theories are driven by human beings. However, as Simonton has argued, random combinations of “mental elements” in the unconscious can lead to highly surprising and creative ideas whose origins remain “unknowable” to the individual [36]. In a similar way, we suggest that heuristics, often operating on the periphery of researchers' consciousness, are applied to research problems and then re-applied when success is achieved. An interesting characteristic deserving further investigation is researchers' ability to retain the same heuristic when moving from one project to another or even from one field to another. This trait reinforces our analogy with the concept of selection, meaning that the environment ultimately determines whether they fit or not.

As our results have demonstrated, high impact researchers act based on a relatively complex network of causal elements. Through our modeling effort we have demonstrated the important role of feedback loops in assisting researchers to choose whether their heuristic fits the needs of the field they have chosen. By performing System Dynamics analyses, previous researchers have been able to optimize systems by shortening the delay in a feedback loop. Based on our models we believe that the same concept can apply to high impact impact systems and its component researchers. In other words, the quicker novice researchers can receive feedback on whether their strategies are aligned with the common practices for a certain scientific group, the quicker they will either be able to refine their heuristic or they will quit science altogether and look for another area where their heuristic might be a better fit.

A final point should be made in relation to the distinction between distributed cognition [45] and the selection mechanisms outlined in our project. Although the concept of distributed cognition, or receiving feedback from the other individuals in a group might bear some resemblance to the selection mechanism we describe, the two concepts are complementary but distinct. While one of our study respondents clearly outlined his main heuristic to be drawn from comments made by his group during conferences held at regular intervals, this heuristic was by no means shared by any of the other researchers in our study. This does not invalidate Dunbar's findings, but it does place limitations in that this is not the prevalent method across researchers or fields. Instead, distributed cognition should be considered as one heuristic, and it should be chosen when the combination of environmental and individual characteristics make it achieve the best practical results.

Despite our results being novel and having implications for training and research policy, our study has limitations. First, we had a small sample size (n = 13), which was primarily influenced by the difficulty in recruiting high impact researchers as a result of their busy schedule. Despite the small sample size, which might not be considered representative, the consistency in themes across researchers points toward a high likelihood that the mechanisms we identified in this study are widely prevalent. Second, our researchers represented a broad range of fields, from translational to clinical and health policy. It is therefore uncertain whether restricting our analyses to more restricted fields might have lead to a narrower group of heuristics. Nonetheless, based on our results even if this were the case not all researchers in a field would necessarily learn the heuristics that might be typical of a given specialty. Instead, a different, stricter type of selection would probably occur. Third, it is believed that working at a successful or prestigious institute/organization enhances the chances of success, which is a bias that was not addressed in this study. Fourth, information like demographics, experience and expertise of the subjects would have further helped in interpreting our results. But these data were not collected in accordance to the IRB regulations and hence were not reported. Finally the study also did not have a comparable comparator group which limits the generalizability of our findings. A followup study with an appropriate comparator group is needed to address this limitation.

In conclusion, our final findings contradicted our initial hypothesis of researchers achieving high impact “by design” or purposefully using methods that would lead to high impact. Instead, we identified a mechanism that bears close resemblance to a selection system. Based on our findings, high impact is not achieved only intentionally, but rather by the presence of a unique skill set that is used by researchers to identify strategic niches and gaps which ultimately results in higher impact. These findings have important implications for researcher training, since rather than attempting to teach novice researchers specific heuristics that we believe would make them successful, training should assist them in finding which unique characteristics they personally have and that could make them unique. This unique characteristic would then give them a competitive advantage and ability to “survive” in a highly competitive environment. In sum, the generation of high impact research is all but a linear and predictable system. Random events constantly happen, and some researchers use their heuristics to capture this randomness and turn it into more high impact findings and discoveries. In natural selection language, this search would be equivalent to a process of speciation [46]. Future studies should be conducted in parallel with training programs so that this mechanism of speciation can be understood in the making, perhaps testing the limits to which the selection metaphor can be pushed to better understand how we should guide the next generation of biomedical researchers.

## Supporting Information

Appendix S1 Final Script.
(0.04 MB DOC)
