# Supplementary material for: Selection Mechanisms Underlying High Impact Biomedical Research - A Qualitative Analysis and Causal Model
Source: PLoS One. 2010 May 7;5(5):e10535. doi: 10.1371/journal.pone.0010535 (PMC2866320; doi:10.1371/journal.pone.0010535)
Supplement: Appendix S1 — Final Script. (0.04 MB DOC) [file pone.0010535.s001.doc]

Appendix   S1

- Identify common factors among the three or four of your publications which, in your opinion, had the most impact
- Perceptions about importance of scientific impact
  - Contribution to knowledge
  - Setting a mark as a researcher
  - Peer influence
- Previous influence
  - Personal drivers
  - Role models
  - Previous education
- Selecting field
  - Main factors leading to that choice
  - Higher chance of impact?
- Foreseeing impact
  - Prior to project
  - Rules of thumb
  - Identifying impact once you see it
  - When? During design, writing, other phases?
- Feasibility
  - Data
    - Availability
    - Infrastructure for collection
    - Personnel
  - Method
    - Familiarity
  - Multiple experiments
- Aesthetic factors
  - Text persuasion
  - Images
- Perspectives and their influence on impact to a certain audience
  - Academic
    - Peer influence
    - Feedback during conferences and presentations
  - Healthcare
    - Providers
    - Policy makers
    - Patients
  - Economic
    - Spin offs
    - Patents
- If you were to look back and provide advice to yourself as a younger researcher, what would you say to him/her?
